# *Bifidobacterium animalis* subsp. *lactis* 420 for Metabolic Health: Review of the Research

**DOI:** 10.3390/nu12040892

**Published:** 2020-03-25

**Authors:** Henna-Maria Uusitupa, Pia Rasinkangas, Markus J. Lehtinen, Sanna M. Mäkelä, Kaisa Airaksinen, Heli Anglenius, Arthur C. Ouwehand, Johanna Maukonen

**Affiliations:** DuPont Nutrition & Biosciences, Global Health and Nutrition Science, Sokeritehtaantie 20, 02460 Kantvik, Finland; pia.rasinkangas@dupont.com (P.R.); markus.lehtinen@dupont.com (M.J.L.); sanna.makela@dupont.com (S.M.M.); kaisa.airaksinen@dupont.com (K.A.); heli.anglenius@dupont.com (H.A.); arthur.ouwehand@dupont.com (A.C.O.); pia-johanna.maukonen@dupont.com (J.M.)

**Keywords:** *Bifidobacterium lactis*, B420, gut microbiota, metabolic health, metabolic syndrome

## Abstract

The growing worldwide epidemic of obesity and associated metabolic health comorbidities has resulted in an urgent need for safe and efficient nutritional solutions. The research linking obesity with gut microbiota dysbiosis has led to a hypothesis that certain bacterial strains could serve as probiotics helping in weight management and metabolic health. In the search for such strains, the effect of *Bifidobacterium animalis* subsp. *lactis* 420 (B420) on gut microbiota and metabolic health, and the mechanisms of actions, has been investigated in a variety of in vitro, pre-clinical, and clinical studies. In this review, we aim to highlight the research on B420 related to obesity, metabolic health, and the microbiota. Current research supports the hypothesis that gut dysbiosis leads to an imbalance in the inflammatory processes and loss of epithelial integrity. Bacterial components, like endotoxins, that leak out of the gut can invoke low-grade, chronic, and systemic inflammation. This imbalanced state is often referred to as metabolic endotoxemia. Scientific evidence indicates that B420 can slow down many of these detrimental processes via multiple signaling pathways, as supported by mechanistic in vitro and in vivo studies. We discuss the connection of these mechanisms to clinical evidence on the effect of B420 in controlling weight gain in overweight and obese subjects. The research further indicates that B420 may improve the epithelial integrity by rebalancing a dysbiotic state induced by an obesogenic diet, for example by increasing the prevalence of lean phenotype microbes such as *Akkermansia muciniphila*. We further discuss, in the context of delivering the health benefits of B420: the safety and technological aspects of the strain including genomic characterization, antibiotic resistance profiling, stability in the product, and survival of the live probiotic in the intestine. In summary, we conclude that the clinical and preclinical studies on metabolic health suggest that B420 may be a potential candidate in combating obesity; however, further clinical studies are needed.

## 1. Introduction

The past decades of research have enabled us to better understand the key role of different microbial populations in human health and disease. Our microbiota is not only remarkable in its abundance, but also in its impact on health, interacting continuously with our body and either sustaining health or causing disease, depending on the ecological function of the microbes [1].

The gastrointestinal tract harbors a vast number of bacteria (10^13^), which roughly equals the number of cells that make up the human body [2]. Commensal gut bacteria are involved in many metabolic processes such as fermentation of undigested carbohydrates into short-chain fatty acids and other metabolites, digestion and absorption of nutrients, but also in the maturation of the immune system, as well as providing protection against incoming, potentially pathogenic microbes. Research indicates that specific probiotic strains or their combinations could be used to restore or maintain the composition and activity to a “healthy” intestinal microbiota and, thus, reduce the risk for a range of diseases or unfavorable conditions [3,4]. Maintaining the ecological balance of the complex microbial community in the gastrointestinal tract has been associated with the development and maintenance of intestinal immune function and metabolic processes, as well as other physiological functions, making the microbiota a critical factor for general human wellbeing [5,6,7].

The origin of a microbial strain or its natural habitat (e.g., the human gastrointestinal tract) is not a guarantee or precondition of its performance as a probiotic from the efficacy, safety, technological, or application perspective. Instead, in addition to being “live microorganisms that, when administered in adequate amounts, confer a health benefit on the host” [8], a probiotic strain should be proven safe by appropriate phenotypic and molecular techniques and/or toxicology studies for acute or chronic toxicity, be able to resist acid and bile to survive the upper gastrointestinal tract, have good technological properties to be produced at large scale, and survive in sufficient counts until the end of shelf life.

Bifidobacteria were discovered at the turn of 18th and 19th centuries by Tissier in the feces of breast-fed infants, and since then, *Bifidobacterium* spp. have been shown to be comprised of Gram-positive, non-spore forming, anaerobic, pleomorphic bacteria [9,10,11]. Bifidobacteria have been shown to represent one of the most abundant genera present in a healthy gut early in life, being the most abundant genus present in the intestine of healthy breastfed infants, and to play an important role in gut homeostasis and immune system development [12,13,14]. During late adulthood and in several diseases, the levels of *Bifidobacterium* spp. and its species diversity have been shown to decrease [15]. In general, and relative to the stage of life, a higher proportion of bifidobacteria in the intestinal tract is considered beneficial to health. Today, evidence has emerged to indicate the impact of many bifidobacteria on the host’s immune system and metabolism, resulting in an association with a range of health benefits such as a reduced risk of respiratory tract infections and various gastrointestinal disorders and infections, particularly antibiotic associated diarrhea [15,16]. The only certain way to establish the true benefit of a probiotic strain is by systematic in vitro and in vivo studies and, in particular, randomized and placebo-controlled human intervention studies.

*Bifidobacterium animalis* subsp. *lactis* (*B. lactis*) is one of the most common *Bifidobacterium* species utilized as a probiotic in commercial products in North America and Europe. *B. lactis* has been used in fermented foods for decades and was scientifically classified by Meile et al. in 1997 [17], then re-classified as *B. animalis* subsp. *lactis* in 2004 [18]. However, for simplicity, we will here refer to the species as *Bifidobacterium lactis*.

One of the probiotic strains that has been studied for its mechanism of action and clinical benefits is *Bifidobacterium animalis* subsp. *lactis* 420 (B420). Recently, the complete genome sequence of B420 has been published, allowing for more stringent strain identity confirmation among other genetically similar *B. lactis* strains [19]. The health benefits that have been shown with B420 consumption include for example control of body fat mass gain in a human intervention trial [20]. Preclinical data furthermore suggest enhancement of mucosal integrity [21,22] and glycemic control [23], as well as improving host resistance to pathogens [24,25]. In this review, we will discuss the preclinical and clinical studies on B420 and the mechanisms of action of the associated health benefits, as well as the technological properties of B420 in the context of an industrial probiotic. The review is based on literature searches performed in PubMed, and the manuscript includes all published data on B420 and metabolic health published prior to February 2020.

## 2. B420 and Health Benefits

To date, probiotic interventions and mechanistic trials related to weight management and metabolic health have mainly focused on the genera *Lactobacillus* and *Bifidobacterium*. B420 has shown promise in weight maintenance in a randomized placebo-controlled clinical study, as well as inducing a better metabolic health state in animal studies via glycemic control by reducing glucose levels and improving insulin sensitivity [20,23,26].

There is growing evidence on the ability of B420 to affect weight and metabolism via gut barrier function and gut microbiota composition modulation in various in vitro and in vivo trials, as well as in a clinical study. An important feature of B420 in weight maintenance can be related to its ability to enhance intestinal epithelial integrity in vitro [22] and in vivo [27]. Furthermore, in obesogenic mouse models, B420 feeding has been shown to decrease in the quantity of inflammatory markers and gut-derived bacteria in tissues [23,25].

The early findings obtained from in vitro and animal studies led to the hypothesis that B420 could reduce metabolic endotoxemia by improving gut barrier function, and hence, its consumption could lead to improvement in metabolic health, and consequently to reduced fat mass. This mechanistic hypothesis has been used in the design of the later in vivo and human intervention trials with B420. Based on current research, it seems that the benefits of B420 on metabolic health are associated with its ability to modulate the complex web of intertwined metabolic pathways.

### 2.1. Gut Microbiota Composition in Obesity

The differences in gut microbiota composition between obese and lean mice and humans was initially reported by Ley et al. (2005) and Turnbaugh et al. (2006) more than a decade ago [28,29]. Since then, early publications in cross-sectional studies about the differences between healthy and obese subjects have indicated that large phyla-level shifts, mainly as an increase in the *Firmicutes*/*Bacteroidetes* ratio, can correlate with obesity [30]. More recently, a higher *Firmicutes*/*Bacteroidetes* ratio of obese children compared to normal weight children has been reported, indicating early discordant shifts in microbial balance in childhood obesity [31,32].

However, there have been discrepancies between these findings, and as Walters et al. (2014) pinpointed in their meta-analysis, methodological differences make the comparison of various studies difficult [33]. Moreover, the methodological differences can start already at DNA extraction, which can yield 10 to 1000 fold differences depending on the method used and bacterial group studied [34]. Methodological variation is not, however, the only factor explaining the observed incoherence. Le Chatelier et al. (2013) showed that the microbiota compositional shifts are present only in certain subpopulations of obese individuals [35]. In these predisposed individuals, it was observed that 36 bacterial genera were less dominant, including *Faecalibacterium*, *Bifidobacterium*, *Lactobacillus,* and *Akkermansia*, among others, and the changes in microbiota composition were associated with metabolic disturbances [35,36]. It has also been shown in some studies that dietary intake correlates more strongly with changes in microbiota than body mass index does [37].

The variability in the diversity of microbial species causes differences between lean and obese individuals’ energy balance by affecting the efficiency of energy harvest, as well as the storage capacity and utilization of the harvested energy [30]. Decreases in resting energy expenditure have been shown to coincide with an increase in the abundance of the *Firmicutes* phylum with a 20% increase corresponding to an increase of 150 kcal in energy harvest per day in humans [38]. Furthermore, it has been suggested that changes induced by probiotics on the microbiome affecting energy metabolism and appetite could shift an individual from a microbiota associated with an obese phenotype to that of a lean one, consequently altering the phenotype as well [39].

As a consequence of the above-mentioned ability of microbes to affect energy metabolism, the most important step in the mode of action of probiotics in metabolic health outcomes might well be inducing a beneficial shift in microbiota composition. The effect of B420 on microbiota composition was studied as part of a placebo-controlled human intervention trial [40]. The results indicated that B420 consumption modulated the gut microbiota—both alone, as well as in synbiotic product containing prebiotic fiber with probiotic—of an overweight study population towards the composition associated with a lean phenotype [40]. B420 alone was shown to increase the relative levels of beneficial microbes, such as *Lactobacillus* spp. and *Akkermansia* spp. [40]. Furthermore, *Bifidobacterium* spp. was positively correlated with lean body mass, a finding that is in accordance with a previous publication reporting significantly less fat mass after B420 consumption compared to placebo [20]. A synbiotic product consisting of B420 together with polydextrose (PDX, a water-soluble branched oligomer of glucose and sorbitol classified as dietary fiber) administered in a human clinical trial [20] showed an increase of the relative proportion of *Akkermansia* spp., Christensenellaceae, and *Methanobrevibacter* spp. in the human fecal microbiota, while the prevalence of *Paraprevotella* spp. was reduced [40]. Christensenellaceae positively correlated with the fecal branched chain fatty acids (BCFAs) [40], which in previous studies have been indicated to inhibit de novo lipogenesis, thus potentially affecting lipid and glucose metabolism in human adipocytes [41]. Further, in the synbiotic group where an increase in the prevalence of Christensenellaceae was seen, this negatively correlated with energy intake, waist-hip-ratio at baseline, waist-area body fat and cholesterol markers [20,40].

### 2.2. Influence of B420 on Weight Management

Amar and colleagues demonstrated in mice that B420 supplementation was able to attenuate fat mass gain in obese and diabetic mice, concomitantly decreasing the translocation of commensal intestinal bacteria into blood and adipose tissue increased by high fat diet (HFD)-induced diabetes [25]. Moreover, in another mouse study, B420 was shown to reduce fat mass accumulation (1.89 g compared to placebo after a six week intervention, *p* = 0.02) in HFD-induced diabetic mice, when supplemented for six weeks as a single strain [23].

In line with these results, it has also been shown in a post hoc factorial analysis of a randomized clinical study that B420 supplementation resulted in significantly less total body fat mass (−4%, *p* = 0.002 vs. non-B420 containing groups, per protocol (PP) population) and waist circumference (−2.4%, *p* = 0.004 vs. non-B420 containing groups, PP population). In addition, the effect seemed to be concentrated in the fat localized in the central region of the body and thus seen as favorable changes in trunk fat mass (*p* = 0.0002) and android fat mass (*p* = 0.004) in the PP population when compared to groups not consuming B420 [20]. Combining B420 with other probiotics, or prebiotics—as synbiotic combination products—offers further possibilities regarding metabolic health. In the previously mentioned clinical trial, a synbiotic consisting of B420 with PDX was able to control body fat mass accumulation after a six month intervention with an average difference of 1.4 kg in total body fat mass (*p* = 0.02) between the synbiotic group and placebo group in the PP population [20]. The difference in body fat mass was most evident in the trunk region where there was 6.7% less fat mass (*p* = 0.008) and a 2.7% (2.6 cm) smaller waist circumference (*p* = 0.047) in the synbiotic group compared to the placebo group at the end of the intervention [20]. As trunk fat accumulation is associated with ectopic fat accumulation, controlling this inner organ fat is crucial for metabolic health. In the context of potentially synbiotic effects, an in vitro study showed that B420 was only weakly able to utilize PDX for growth compared to other complex oligosaccharides, xylo-oligosaccharide (XOS), fructo-oligosaccharide (FOS), or galacto-oligosaccharide (GOS) [42]. This enables sustained fermentation of PDX throughout the colon, but also indicates that intestinal survival of B420 is likely not dependent on the presence of PDX as a substrate.

Furthermore, in the human intervention trial, B420 significantly reduced energy intake by approximately 210 kcal/day (*p* = 0.037) compared to the non-B420 containing group [20]. This finding was supported by the earlier in vitro findings, in which the expression of satiety marker peptide YY (PYY) was shown to be increased by B420 [43]. The role of the intestinal microbiota in host appetite and food intake has been suggested to be conveyed through both regulation of eating-related behavior, possibly via the microbiota gut-brain axis [44], as well as via directly acting on molecules regulating appetite and satiety [45]. Therefore, some of the effects on metabolic health observed in clinical trials with B420 might be a result of changes in satiety and appetite hormone levels affecting food intake. However, this hypothesis requires further validation.

### 2.3. Influence of B420 on Glycemia, Lipidemia, Insulin Sensitivity, and Cardiovascular Disease Risk

**Figure 1:**
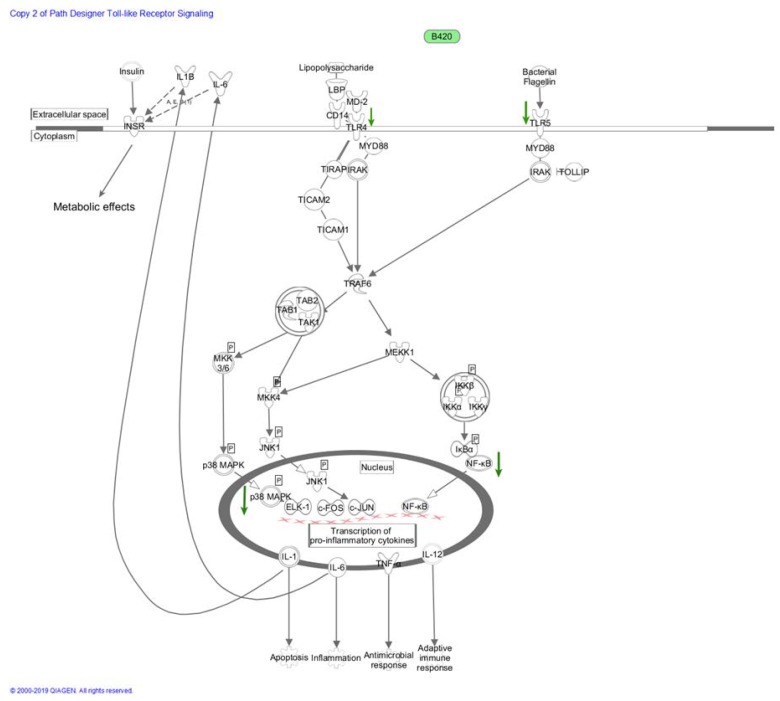
Schematic proposal of how B420 and/or its metabolites presumably affect cellular signaling via downregulation of TLR pathways, indicated as light green arrows in the figure, as well as the inflammatory IKKα-NF-κB pathways or p38 MAPK-ELK-1 pathways. B420 has been shown to enhance epithelial integrity in vitro [22] and to decrease the levels of circulating LPS in mice [27]. LPS triggers inflammation through upregulation of IKKα-NF-κB pathways and p38 MAPK-ELK-1 pathways [72], indicated as dark green arrows in the figure. Downregulation of these pathways by B420 reduces the transcription of pro-inflammatory cytokines in the nucleus. As proinflammatory cytokines are excreted out of the cell, and affect insulin receptor activity, this cascade can serve as one mechanistic route via which B420 exerts its metabolic effects through affecting epithelial integrity and cytokine levels. LPB = lipopolysaccharide binding protein; MD-2 = lymphocyte antigen 96, a protein associated with toll-like receptor 4; CD14 = cluster of differentiation 14; TLR4 = toll-like receptor 4; MYD88 = myeloid differentiation primary response protein 88; TIRAP = toll-interleukin 1 receptor (TIR) domain containing adaptor protein, IRAK = interleukin-1 receptor associated kinases; TICAM2 = toll-like receptor adaptor molecule 2; TICAM1 = toll-like receptor adaptor molecule 1; TRAP6 = thrombin receptor activator peptide 6; TAB2 = TAK1-binding protein 2; TAB1 = TAK1-binding protein 1; TAK1 = TGF-β activated kinase 1; MKK3/5 = mitogen-activated protein kinase kinase 3/5; p38 MAPK = p38 mitogen-activated protein kinase; MKK4 = mitogen-activated protein kinase kinase 4; JNK1 c-Jun N-terminal kinase 1; ELK-1 = ETS-like gene 1 (coding for ETS like protein Elk-1); c-FOS = Fos proto-oncogene, which is an AP-1 transcription factor subunit; c-JUN = Jun proto-oncogene, AP-1 transcription factor subunit; NF-κB = nuclear factor kappa B; TNF-α = tumor necrosis factor alpha; IL-12 = interleukin 12; IL-6 = interleukin 6; IL-1 = interleukin 1; MEKK1 = mitogen-activated protein kinase kinase kinase 1; IKKβ = I kappa B kinase beta; IKKα = I kappa B kinase alpha; IKKγ = I kappa B kinase gamma; TLR5 = toll-like receptor 5; TOLLIP = toll interacting protein; IL1β = interleukin 1β; INSR = insulin receptor. The schematic networks were generated through the use of IPA (QIAGEN Inc., https://www.qiagenbioinformatics.com/products/ingenuity-pathway-analysis).

Chronic overnutrition eventually leads to hyperlipidemia and hyperglycemia, which in turn, can cause insulin resistance via activation of stress-response and inflammatory signaling pathways [46]. Hormonal regulation of carbohydrate and other energy-rich nutrient metabolism is closely intertwined, and as previously stated, gut microbiota composition can affect the energy harvesting capacity by, e.g., modulating the number and affinity of transporter receptors [29]. Binding of insulin to its receptors normally results in the translocation of glucose transporter 4 (GLUT4) to the plasma membrane and glucose absorption (Figure 1), but also to upregulation of lipogenic activity [47,48]. The biochemical abnormalities in the diabetic state trace back to reduced entry of glucose, as well as to overaccumulation of lipids [49].

In the resting state of the liporegulatory system, when caloric intake is equal to expenditure, lean tissues contain little or no unmetabolized lipids. Positive energy balance promotes an increased mass of adipose tissue by hypertrophy (increased adipocyte cell size) and hyperplasia (increased adipocyte cell number), to buffer the effects of surplus energy intake on lean tissues. Hyperplasia promotes the secretion of antiobesity hormones such as leptin. Leptin was initially identified as a satiety hormone involved in energy balance by regulating fat storage, but it has also been shown to be involved in immune responses via modulating cytokine Th1/Th2 balance and promoting inflammatory response [50]. Hyperplasia enhances lean tissue oxidation of surplus lipids through downregulation of lipogenic enzymes [51,52]. Moreover, these events activate fatty acid beta-oxidation, further increasing the oxidation of surplus fatty acids [53]. Adipocytes have likely developed to buffer plasma fatty acid concentration by storing large quantities of triacylglycerols as non-specialist cells [52]. However, in insulin resistance, delayed hyperinsulinemia increases ectopic fat accumulation, closing the vicious cycle of ectopic fat accumulation and impaired glucose tolerance [54]. Leptin is involved in modulation of inflammation through the T cell compartment, forming a link between excessive fat accumulation and various inflammatory states [50].

Since the associations between gut microbiota composition and metabolic health have been observed, probiotics have been suggested as a potential therapeutic tool to improve insulin sensitivity. Promising indications have been obtained from a study by Vrieze et al. (2012), in which a fecal microbiota transplant from a lean donor improved insulin sensitivity in men with metabolic syndrome, indicating the ability of gut microbiota modification to restore impaired glucose intolerance [55].

The ability of B420 to support weight management has been shown to be associated with an attenuation in the progression of metabolic health disorders in dietary mouse models of diabetes and obesity [23,25]. In a diabetes mouse model study, B420 normalized the insulin sensitivity and fasting hyperinsulinemia with the fasting blood glucose of the B420 + HFD group (6.9 mM) being at the same level as that of a normal chow diet group (6.7 mM) and significantly lower than in the HFD alone group (8.2 mM, *p* < 0.05), and similar results were obtained from the glucose turnover rate. Additionally, positive effects were seen in tissue inflammation as the expression of major proinflammatory cytokines, interleukin (IL)-6, IL-1β, and plasminogen activator inhibitor (PAI)-1, was reduced (*p* < 0.05) in mesenteric adipose tissue [25].

Further, the gut microbiota also seems to play a role in the progression of cardiovascular diseases. So far, a direct association between the severity of myocardial infarction and gut microbiota composition has been shown in mice [56]. Moreover, in a recent study by Danilo et al. (2017), B420 mitigated the pathological impact of myocardial infarction in a mouse model [57]. In this study, myocardial infarction was induced in mice by an ischemia/reperfusion method after pre-treatment with either placebo, B420, or *Lactobacillus salivarius* Ls-33 [57]. Pretreatment with B420 for four weeks attenuated the cardiac injury by reducing significantly (*p* < 0.05) the infarct size and area when compared to saline-treated mice and a quenched inflammatory transcriptional profile resulting in lower levels of inflammatory markers such as IL-6 in the infarct area [57]. The observed associations can be mediated by the microbial metabolites interacting with cell surface receptors such as kinases containing ion channels located on the heart cell surface [58]. Lam et al. (2016) suggested that the observed beneficial effects of probiotics on myocardial infarction are due to low molecular weight metabolites, such as phenylalanine, tryptophan, and tyrosine metabolites, produced by the intestinal microbiota, which affect protein kinases and potassium channels in signal transduction pathways [58]. Therefore, clinical studies to further elaborate the ability of B420 to alleviate myocardial infarction are warranted. Moving forward, it would be interesting to study how B420 functions to direct the metabolism of the microbes in a complex environment such as the gastrointestinal tract.

A benefit for combining B420 with antidiabetic drugs has been proposed based on a mouse study [26]. Whereas a low dose of metformin alone reduced plasma insulin concentration, the probiotic showed a similar effect to antidiabetic drug by lowering plasma glucose levels (B420 alone: 9.77 mmol/L, metformin with B420: 10.3 mmol/L, control: 10.8 mmol/L, metformin: 11.4 mmol/L, *p* = 0.02) and improving glucose regulation (B420 alone: AUC 2140 mmol/L*min, metformin + B420: 2160 mmol/L*min, control: 2340 mmol/L*min, metformin: 2660 mmol/L*min, *p* = 0.002 [26].

Furthermore, in the same study, a synbiotic product including B420 with PDX showed benefits for glycemic response and fasting plasma glucose in mice. Fasting glucose was not affected by sitagliptin, a medication used to treat diabetes mellitus type 2, whereas B420 both alone and in combination with PDX induced a statistically significant decrease [26]. Similarly, Garidou et al. obtained interesting results in a mouse model in which a type 2 diabetes resembling state with glucose intolerance, insulin resistance, and dysbiosis in the gut microbiota was induced by HFD [59]. The HFD-induced microbiota dysbiosis caused a decrease in the numbers of IL-17/RORᵧt T cells and Treg cells in the small intestinal lamina propria, but when the HFD-fed mice were given B420 with PDX as a synbiotic treatment, the T cell numbers were similar to the level of the normal chow-fed mice [59]. Further, the fasting glycemia of synbiotic and HFD-fed mice was lower (4.9 mM) than that of mice receiving only HFD (7.5 mM), and similar to that of conventional normal chow-fed mice [59]. Furthermore, in a placebo-controlled, double-blind, randomized crossover trial, in which B420 was administered as a probiotic combination including *Lactobacillus acidophilus* 74-2 for a five week intervention period, the combination affected positively plasma lipid profile in healthy adults [60]. The concentration of triacylglycerols decreased significantly by 11.6% (*p* = 0.045) during the probiotic period, but no changes were detected in cholesterol levels, which might be due to the short intervention period [19].

### 2.4. Metabolic Endotoxemia and Chronic Low-Grade Inflammation in Gut Dysbiosis

Gut dysbiosis refers to a state of microbial imbalance caused by perturbations in the structure or functions of the microbial communities [61,62]. Microbiota disruption can result in the loss of beneficial microbes, reduced diversity or pathobiont expansion, with normally dominating species becoming underrepresented and outcompeted by atypical organisms.

An inflammatory response is a complex self-limiting process coordinated by vasoactive amines, adhesion molecules, lipid-derived eicosanoids, cytokines, and chemokines. Inflammation is fundamentally a protective mechanism. However, when the self-limiting nature of this process is inappropriately regulated, it is transformed into a detrimental, chronic state of inflammation, often referred to as chronic low-grade inflammation, which multiple studies have indicated to play a crucial role in metabolic disorders [63].

The dysbiosis paradigm recognizes the interrelations of gut microbiota and metabolic health in more detail and is based on the idea that gut microbiota composition can affect intestinal barrier function and thus regulate the translocation of inflammatory gut microbes and their components, which then cause tissue inflammation by affecting immunomodulatory metabolic pathways [64]. Obesity has been shown to be associated with increased gut permeability both in animal [65], as well as in human studies [66].

Amar and colleagues (2011) demonstrated in mice that translocation of commensal intestinal bacteria into blood and adipose tissue is increased during the onset of HFD-induced diabetes [25]. Moreover, the translocation results in low-grade bacteremia, and the presence of viable bacteria in the blood is further associated with CD14, a lipopolysaccharide binding protein, functioning as the endotoxin receptor, and Nod1, a receptor recognizing bacterial molecules and inducing immune responses, and is regulated by adipokine leptin. A one month treatment with B420 reduced the mucosal adherence of *Escherichia coli* and bacterial translocation of Enterobacteriaceae into adipose tissue, thus reversing the bacteremia [25]. Furthermore, the expression of the major pro-inflammatory cytokines—TNF-α, IL-1β, and IL-6, and coagulation regulator PAI-1—was reduced in mesenteric adipose tissue, liver, and muscle by B420 and associated with positive changes in insulin sensitivity, as described in more detail earlier in this review [25].

Metabolic endotoxemia refers to the hypothesis in which microbes [25], or microbial fragments, such as lipopolysaccharide LPS [67], peptidoglycan [68], and flagellin [69], enter the bloodstream from the gut and end up in different tissues, causing exaggerated lipolysis and low-grade inflammation. In an in vivo study, treatment with B420 decreased bacterial adherence to the intestinal mucus of mice [23]. Furthermore, in vitro findings from cell culture studies indicated the superiority of B420 among the screened strains in enhancing epithelial integrity in an intestinal epithelial cell model [21,22].

Later mice studies verified that similar results could be obtained in vivo, and B420 was shown to reduce epithelial translocation of *E. coli*, as well as to lower the circulating LPS levels in two separate study settings [23,25]. Once in circulation, LPS binds to LPS binding protein (LBP), activating the CD14 receptor and further the toll-like receptor 4 (TLR4) [70]. As TLR4 is connected to insulin metabolism via cytokine signaling, activation of TLR4 links LPS to insulin resistance presumably by altering insulin receptor signaling in the presence of inflammatory cytokines [71], as explained in more detail in Section 2.5.2. and illustrated in Figure 1. In addition, the interrelations of cellular signaling pathways, cytokines related to inflammatory responses, and insulin metabolism are illustrated in Figure 1.

In a clinical study, B420 appeared to keep the levels of circulating zonulin, a potential marker of intestinal permeability, consistently lower throughout the study compared to groups without B420 [20]. Furthermore, changes in inflammation marker high sensitivity C-reactive protein (hsCRP) were significantly correlated with the changes in zonulin, although they did not reach statistical significance as such [20]. The clinical results support the earlier preclinical findings that B420 improves epithelial barrier function.

### 2.5. Immunomodulatory Pathways in Metabolic Endotoxemia

The mechanism by which the microbes or their fragments translocate from the gut in metabolic endotoxemia is unclear, but it is thought that the integrity of the epithelial layer in the intestinal wall is compromised and the permeability is increased, which leads to translocation of luminal components [25,73,74]. It has been noted that, for example, dietary or antibiotic-induced modification of gut microbiota might lead to a reduction of inflammation and intestinal permeability [73,74]. The consumption of probiotics might also have similar effects, and B420 has been shown to improve epithelial barrier function in cell culture [21,22] and to reduce the epithelial translocation of *E. coli*, as well as circulating LPS levels in mice [23,25]. It is likely that this effect is induced also by other mechanisms than solely the modification of gut microbiota composition.

Immunomodulatory pathways refer to linked signaling pathways that are responsible for communicating signals by various agents and particles to different parts of the body to induce required responses. The connection between diet and intestinal permeability has been commonly accepted, and for example, the effect of dietary carbohydrate composition on postprandial hyperglycemia and postprandial insulin response is well established [75]. However, it is far more controversial how the signaling pathways serve as mechanistic linkages between diet, gut microbiota, and metabolic health delivering these effects. Lately, the ability of food-like components to alter postprandial and fasting state metabolism through modulation of various signaling pathways has been proposed. For example, the AMPK (adenosine monophosphate activated protein kinase)-SIRT1 (silent information regulator T1) cascade serves as an indicator of cellular energy status and is thus an important regulator of carbohydrate and fat metabolism [76]. As B420 has shown promising effects in many animal and in vitro trials related to metabolic health, modulation of these signaling pathways by B420 can certainly be considered as a potential mechanism mediating the observed effects and is worth further investigation (summarized in Figure 1).

#### 2.5.1. Cyclooxygenase and Nitric Oxide Synthase Pathways

Cyclooxygenases (COX) are enzymes functioning in the rate-limiting step of the arachidonic acid cascade to form eicosanoids, thromboxanes, and prostaglandins, which can mediate vasoconstriction or inflammatory functions depending on the eicosanoid, receptor type, and distribution. In eukaryotic cells, two COX isoforms exist: COX-1, which is involved in the maintenance of the physiological functions in a constitutive way, whereas COX-2 as an inducible enzyme mediates mitogenic and inflammatory responses, even though continuous discussion exists about the exact roles of the two isoforms [77]. COX-2 is generally present in low levels in mammalian tissues, unless induced by one of many types of stimuli such as growth factors and cytokines [78].

Arachidonic acid is released from membrane phospholipids through phospholipase A_2_ cleavage and can be metabolized through the COX pathway into prostaglandins and thromboxane A_2_, or by the lipoxygenase pathway to hydroxy- and hydroperoxy-eicosatetraenoic acids and leukotrienes [79]. Inhibition of COX, lipoxygenase and phospholipase A_2_ by for example plant secondary metabolites has been shown in many studies and results in lower circulating levels of these inflammatory eicosanoids in vitro [80], but the idea of probiotics inhibiting these enzymes is rather recent. In an animal study, treatment with a strain of *B. lactis* was able to suppress COX-2 expression and colonic TNF-α production in a trinitrobenzene-sulfonic acid-induced model of rat colitis [81]. Furthermore, in a rat model, B420 supplementation protected from an NSAID-induced increase in gastric permeability [27].

B420 has been shown to affect the COX pathway by producing metabolites that have been observed to upregulate COX-1 in an undifferentiated and differentiated human intestinal epithelial cell model, Caco-2, and concomitantly, downregulate the expression of COX-2 [22,82]. This function is similar to and has previously been elicited by butyrate and propionate, two well-known beneficial microbial metabolites [82]. This effect seems to be a species- and strain-dependent phenomenon, as several other bifidobacteria or lactobacilli share this COX gene regulating effect elicited by B420 [22,82]. In metastatic gastric adenocarcinoma cells, on the other hand, B420 was not able to regulate either COX-1 or COX-2 [83], which indicates that the regulation is cell type-dependent. B420 metabolites were also able to counteract the tight junction integrity-decreasing effect of *E. coli* O157:H7, which has an opposite, COX-2 upregulating and COX-1 downregulating effect [22]. However, the role of eicosanoids in the barrier regulation of the intestinal epithelial cell model by B420 is currently unknown.

The nitric oxide synthase (NOS) pathway is important in the maintenance of bodily functions; endothelial nitric oxide synthase (eNOS) has an important role in maintaining blood pressure homeostasis and vascular integrity, while inducible nitric oxide synthase (iNOS) invokes an inflammatory process [84]. During the past decade, the significance of sustained high NO production by iNOS in intestinal inflammation and gastrointestinal immunopathology, such as chronic inflammatory bowel disease, has become evident [85,86]. The gut microbiota has been shown to regulate circulating amounts of iNOS via microglia activation [87]. Probiotic strains from both genera *Bifidobacterium* and *Lactobacillus* have been shown to possess eNOS and iNOS inhibition, respectively [88,89]. In a study by Putaala et al. (2010), intact bacterial cells of B420 were shown to induce the expression of iNOS via activation of transforming growth factor beta-activated kinase 1 (TAK1) and activator protein 1 (AP1), whereas the cell-free supernatant induced AP1 and inhibited TAK1 [90], indicating differential regulation by cells and bacterial metabolites. This is probably very important since bacterial cells should remain in the lumen of the healthy gut. In animal study designs, *B. lactis* treatment was able to reduce iNOS synthase expression and colonic TNF-α production in a trinitrobenzene-sulfonic acid-induced model of rat colitis [91]. More recently, a probiotic cocktail containing *B. lactis* (strains not reported) among three other probiotics (*L. acidophilus, Lactobacillus plantarum*, and *Bifidobacterium breve*) was shown to promote recovery from acute colitis via inhibition of iNOS, as well as the nuclear factor (NF)-κB pathway [86].

#### 2.5.2. NF-κB and MAPK Pathways

Metabolic endotoxemia is associated with altered cytokine balance in serum favoring proinflammatory cytokine production (such as IL-6, IL-8, TNF-α) over that of anti-inflammatory cytokines (such as IL-10, TGF-β, IL-4). Interfering with this finetuned balance may induce overly active proinflammatory cytokine production in all tissues [46]. Obesity-induced inflammation is partly due to toll-like receptor (TLR) activation [92]. TLRs are innate immune receptors on the cell surface or in the intracellular membranes that recognize various microbe-derived molecules, such as bacterial lipoteichoic acid (TLR2), LPS (TLR4), flagellin (TLR5), and CpG DNA (TLR9), among many others. The activation of TLRs via different adaptor proteins leads to the activation of complex signaling pathways that result in the activation of cytokine gene transcription to induce a proper innate immune response to fight the pathogen. The main signaling pathways coordinating TLR signaling are interferon regulatory factors (IRF), the mitogen-activated protein kinase (MAPK) pathway, and the NF-κB pathway, the latter functioning as the central valve of many chemokines, adhesion molecules, growth factors, acute-phase proteins, cell proliferation, iNOS, invasion, migration, and immune receptors, making it a potent anti-inflammatory target [93].

Not surprisingly, many health-promoting food components such as polyphenols have been shown to exert their functions via inhibition of the NF-κB pathway [94], and recently, the ability of probiotics or gut microbiota metabolites to regulate the NF-κB pathway has gained interest. Overall, there is emerging evidence that probiotics are able to modulate innate pathogen sensing signaling pathways, as reviewed recently by Llewellyn and Foey [95]. However, to date, the data are rather sparse as the responses may be either inhibiting or activating, depending on the probiotic strain, cell/tissue type, experimental setup, and which components of the signaling pathways were analyzed. In an in vitro study by Putaala et al. (2010), the cell-free metabolites of B420 were shown to have to some extent opposite effects on known NF-κB pathway regulator gene expressions than enterohaemorrhagic *E. coli* O157:H7 (EHEC) [90], a pathogenic bacterium known to induce inflammation in intestinal epithelial cells in vitro [22]. Thus, these results can be interpreted to indicate that downregulation of NF-κB pathway presents one possible route through which B420 could affect TLR signaling.

The mitogen-activated protein kinases (MAPK) are a chain of sequentially activated protein kinases that regulate many different cell processes [96]. The MAPK family consists of Ser/Thr kinases including at least extracellular signal-related kinases (ERK)1/2, c-Jun amino-terminal kinases (JNK) 1/2/3, p38-MAP kinase, and ERK5 kinases, of which the JNK and p38 cascades are most involved in inflammation [97]. Cell-free metabolites of B420 were shown to downregulate p38 and ERK in an in vitro study assessing the transcriptional response of human intestinal epithelial cells to various probiotics [90], indicating the ability to influence host cytokine levels via affecting the complex signaling pathways that coordinate cytokine production.

One interesting target for probiotic modulation is TLR4 signaling, which is central in metabolic endotoxemia [98] and linked to obesity, as TLR4-deficient mice are resistant to HFD-triggered obesity [99]. Several lactobacilli strains have been shown to regulate TLR4 signaling negatively such as *Lactobacillus casei* OLL2768 via inhibiting the NF-κB and p38 pathways and upregulating negative regulators Tollip and Bcl-3 in bovine intestinal epithelial cells stimulated with heat-killed enterotoxigenic *E. coli* (ETEC) [100] and *Lactobacillus amylovorus* DSM 16698^T^ by suppressing the ETEC-induced activation in the human intestinal Caco-2/TC7 cell line [101]. Similarly, B420, as well as its cell-free metabolites downregulated TLR4 gene expression in intestinal epithelial cells [90]. However, in a human trial, daily consumption of B420 did not lower IL-6 cytokine levels in overweight and obese adults who had normal range levels at baseline [20], indicating that further research is needed to elaborate these mechanisms in more detail with B420. Intriguingly, mice lacking TLR5, the receptor for bacterial flagellin, developed metabolic syndrome, and their gut microbiota was altered [69]. As B420 decreased TLR5 gene expression in intestinal epithelial cells [90], it would be of great interest to study whether downregulation of TLR signaling and subsequent suppression of inflammation comprise one of the mechanisms by which beneficial microbes may reduce metabolic syndrome and obesity. The proposed mechanistic route through which B420 could affect metabolic health via TLR4 signaling is summarized in Figure 1.

In the search for effective and safe solutions for the worldwide obesity epidemic, B420 has arisen to provide warranted benefits without safety concerns. The ability of B420 to adhere to intestinal mucosa is the basis of all observed beneficial effects. Considering the results above, it seems possible that B420—both alone and as part of synbiotic products—may have a positive modulatory effect on gut microbiota composition by increasing the relative abundance of other bacterial genera with known beneficial effects in the gut. Further, it seems that B420 is able to affect gut microbiota composition in an anti-obesogenic manner by increasing the prevalence of lean phenotype microbes such as *Akkermansia muciniphila.*

## 3. Conclusions 

All in all, it seems evident that B420 has significant beneficial effects on weight management and metabolic health mediated by a complex signaling pathway network yet to be fully understood by current research. The research aiming to understand the impact of B420 on the mechanisms both inside the gut, including mucosa and sub-mucosa (epithelial barrier function), as well as the gut immune system, and at a systemic level has been well initiated. In the future, it will be extremely intriguing to dig deeper into the world of systemic metabolism utilizing machine learning approaches to filter through these complex pathways and find meaningful correlations. Bacterial metabolites have been suggested to be in part responsible for the observed effects of probiotics in general. Future research should also focus on examining the health benefits these bacterial metabolites can have in human.
